# Adult Primary Hepatic Sarcoma Presenting as a Chronic Expanding Hematoma

**DOI:** 10.70352/scrj.cr.25-0811

**Published:** 2026-03-14

**Authors:** Takako Yamada Fujii, Takuya Kimura, Shinichi Nakatsuka

**Affiliations:** 1 Department of Liver Surgery, Yao Tokushukai Medical Hospital, Yao, Osaka, Japan; 2 Department of Pathology, Yao Tokushukai Medical Hospital, Yao, Osaka, Japan

**Keywords:** primary hepatic sarcoma, undifferentiated sarcoma, rupture, chronic expanding hematoma

## Abstract

**INTRODUCTION:**

Primary hepatic sarcoma is extremely rare in adults and can be difficult to distinguish from benign hemorrhagic lesions on imaging. We report a rare case of undifferentiated sarcoma (US) that initially mimicked chronic expanding hematoma (CEH) and ruptured.

**CASE PRESENTATION:**

A woman in her 50s presented with sudden-onset abdominal pain caused by rupture of a hepatic lesion diagnosed radiologically as CEH and underwent right hepatectomy. Preoperative imaging demonstrated a well-circumscribed hypodense mass with rich arterial vascularity and MRI findings suggestive of mixed old and recent hematoma. Nine months later, rapidly progressive peritoneal dissemination developed and was surgically evacuated. Histopathological examination of the disseminated lesions revealed US, and retrospective review of the initial specimen identified similar spindle-shaped tumor cells beneath the fibrous capsule.

**CONCLUSIONS:**

This case illustrates that hepatic lesions resembling CEH may represent or develop into US. Rupture and atypical clinical progression should raise suspicion for malignancy, and careful histopathological assessment of subcapsular tissue is essential.

## Abbreviations

AS
: angiosarcoma
CEH
: chronic expanding hematoma
PHS
: primary hepatic sarcoma
UES
: undifferentiated embryonal sarcoma
UHS
: undifferentiated hepatic sarcoma
UPS
: undifferentiated pleomorphic sarcoma
US
: undifferentiated sarcoma

## INTRODUCTION

Chronic expanding hematoma (CEH) is a rare entity characterized by a fibrously encapsulated hematoma that progressively enlarges due to recurrent hemorrhage and chronic inflammatory changes within the capsule.^1)^ Here, we describe an extremely rare case of a primary hepatic sarcoma (PHS) that initially manifested as CEH with rupture and, following surgical liver resection, recurred with rapidly progressive peritoneal dissemination.

## CASE PRESENTATION

**Fig. 1:**
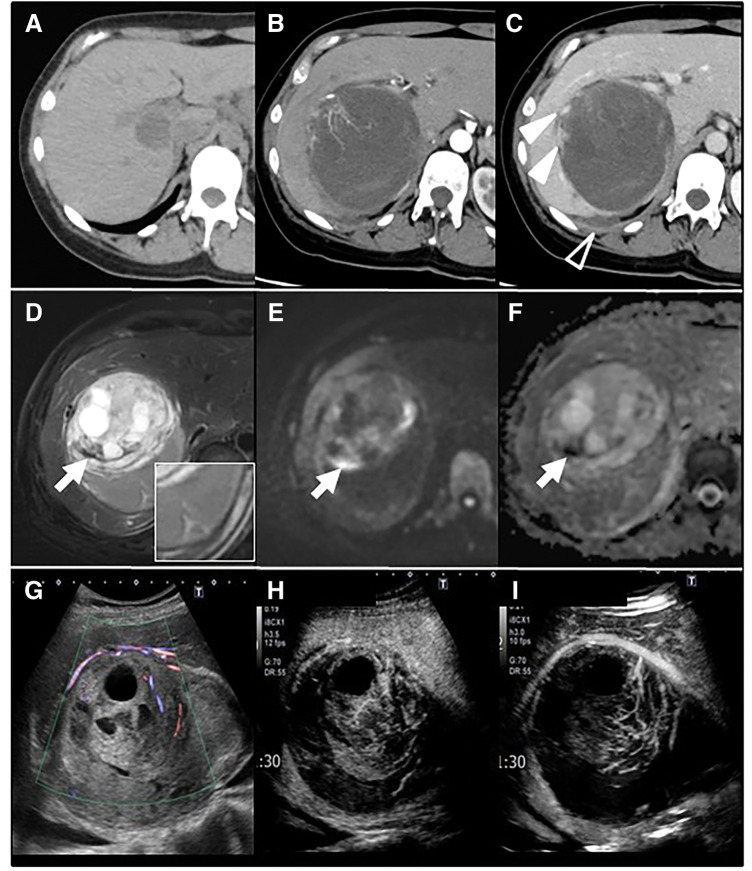
Multimodal imaging findings of the hepatic lesion. (**A**) A plain CT performed 2 years earlier revealed a 1.5 × 2 cm lesion in segment 6 of the liver. (**B**, **C**) A heterogeneous 11.5 cm low-density mass was identified, accompanied by high-attenuation extrahepatic fluid suggestive of bloody ascites (open arrowhead). Perinodular rich arterial vascularity was observed (**B**), and focal enhancement within the mass (solid arrowhead) persisted into the delayed phase (**C**). (**D**) T2-weighted MRI demonstrated a heterogeneous mass encapsulated by a hypointense rim. Very low–signal-intensity components (arrow) indicated hemosiderin deposition, corresponding to areas of diffusion restriction on (**E**) DWI and (**F**) ADC. (**G**) Contrast-enhanced ultrasonography revealed a basket-like pattern with several dilated feeding vessels within the septum. (**H**, **I**) In the early vascular phase, (**I**) the nodule showed no distinct enhancement compared with the surrounding liver parenchyma, followed by early washout. ADC, apparent diffusion coefficient; DWI, diffusion-weighted imaging

A female patient in her 50s felt sudden, severe abdominal pain, and was transferred to our emergency room. She had no significant medical history or previous trauma; however, a hepatic cyst had been incidentally noted on CT 2 years earlier (**Fig. 1A**). Except for mildly elevated hepatobiliary enzymes and slight increases in WBC and CRP, no remarkable findings were observed. Laboratory findings suggested normal hepatic reserve, and no elevation of tumor markers was noted (**Table 1**).

**Table 1:** Laboratory data at admission, with underlined values indicating abnormal results

Complete blood count		Chemistry	
WBC	100 × 10^2^/μL	TP	6.7 g/dL
Neu	59.4%	Alb	3.7 g/dL
Lym	32.8%	AST	37 U/L
RBC	407 × 10^4^/μL	ALT	30 U/L
Hb	12.4 g/dL	ALP	218 U/L
Plt	32.0 × 10^4^/μL	ALP2	72.5%
Coagulation		γ-GTP	119 U/L
PT	110.0%	T-Bil	0.6 mg/dL
PT-INR	0.95	LDH	183 U/L
APTT	27.3 秒	AMY	85 U/L
Fibrinogen	479 mg/dL	BUN	14.8 mg/dL
D-Dimer	2.2 μg/mL	Cre	0.78 mg/dL
Hepatitis virus		CRP	1.15 mg/dL
HCV Ab	(–)	BNP	5.8 pg/mL
HBs Ag	(–)	Glu	120 mg/dL
HBs Ab	(–)	HbA1C	5.6%
HBc Ab	(–)	Fibrosis makers, ICG, Others	
Tumor makers		ALBI score	−1.75
AFP	<10.0 ng/mL	FIB-4 Index	0.80
DCP	19 mAU/mL	M2BPGi	0.34
CEA	2.8 ng/mL	ICG R15	3.6%
CA19-9	5.68 U/mL	ICG K	0.169
		ICG Krem	0.076
		ANA	<40
		AMA	<20

AMA, antimitochondrial antibody; ANA, antinuclear antibody; APTT, activated partial thromboplastin time; ICG K, plasma disappearance rate; ICG Krem, remnant rate; ICG R15, indocyanine green retention rate at 15 minutes; PT, prothrombin time; WBC, white blood cell count

Contrast enhanced CT revealed an 11.5 cm well-circumscribed low-density nodule, along with high-attenuation fluid in Morison’s and Douglas pouch, suggestive of bloody ascites. A dilated and rich neovascularity from A7 hepatic artery developed perinodularly, and the focal enhancement within the mass persisted into the delayed phase.

T2-weighted MRI revealed a heterogeneous, separated mass encapsulated by a thick fibrous rim. Very low-intensity components may indicate hemosiderin deposition, which means it occurs recurrent bleeding and is compatible with findings of diffusion limitation in diffusion-weighted imaging (DWI) and apparent diffusion coefficient (ADC). Contrast-enhanced ultrasonography revealed abundant blood flow around the nodule and in the septum. In the early phase, nevertheless, the nodule itself showed no distinct enhancement compared with the surrounding liver parenchyma, and the enhancement did not persist in the parenchymal area (**Fig. 1**).

Due to the ruptured nodule with very rich vascularity mimicking CEH, we proceeded with surgical resection to control bleeding and achieve a definitive diagnosis. Intraoperative findings represented bloody ascites and a soft tumor with a ruptured capsule, mainly in the posterior segment of the liver. A right lobectomy was performed with an operative time of 3 hours and 40 minutes and a blood loss of 660 mL, without the need for transfusion. The specimen weighed 1002 g, and her postoperative course was uneventful, with discharge on POD 11.

**Fig. 2:**
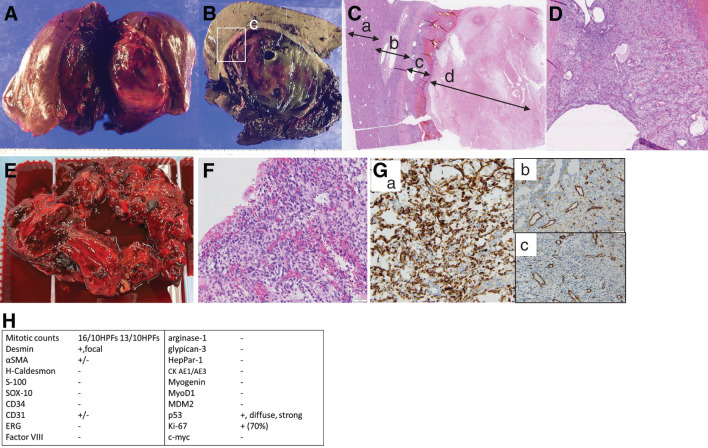
Gross and histopathological findings of the resected specimen. Panels (**A**–**D**) present the pathological findings of the hepatic lesion, and panels (**E**–**H**) present those of the peritoneal dissemination. (**A**) An elastic, soft tumor with a ruptured capsule, predominantly located in the posterior segment. (**B**) A mass consisting of a thick fibrous rim, abundant granulation tissue, and a hematoma with mixed chronic and acute components. The area outlined by the box in (**B**) is shown at a higher magnification in (**C**). (**C**) Histopathological findings of the hepatic lesion (H&E staining); a: Non-neoplastic liver tissue; b: Fibrous capsule; c: Granulation tissue; d: Necrotic and hemorrhagic areas. (**D**) Granulation tissue (H&E staining, ×200). A region of increased cellularity. (**E**) The soft tumor mixed with hematomatous material evacuated from the peritoneal cavity. (**F**) Histologic findings of the peritoneal disseminated tumor (H&E staining, ×200). Highly cellular areas composed of spindle cells with marked architectural and nuclear atypia are observed. (**G**) Immunohistochemical staining. a: Desmin (×200), showing positive tumor cells; b: CD31 (×200), c: α-SMA (×200), b and c show focally weak staining. (**H**) Summary of immunohistochemical results. αSMA, alpha-smooth muscle actin; ERG, erythroblast transformation-specific-related gene

Microscopic examination revealed that the majority of the lesion consisted of a hematoma with mixed chronic and acute components, encapsulated by a thick fibrous wall and containing abundant granulation tissue with capillary-rich vessels. These findings were consistent with a diagnosis of CEH (**Fig. 2A**–**2C**).

**Fig. 3:**
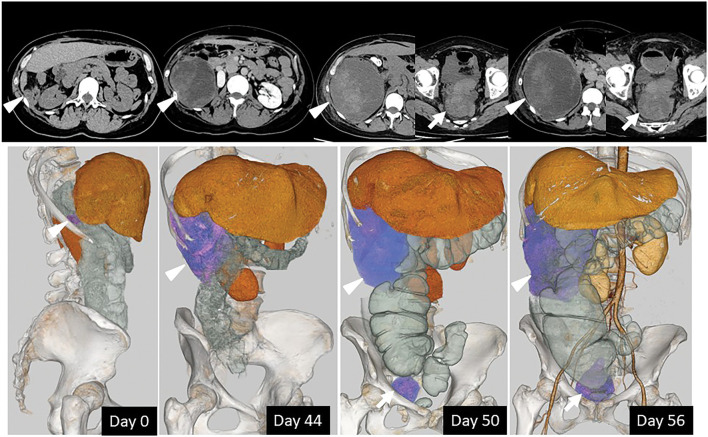
Abdominal CT and 3D reconstructed images of disseminated lesions. The arrowheads indicate the peritoneal lesion along the right paracolic gutter, and the arrows indicate the lesion in the pouch of Douglas; the same symbols correspond to identical lesions on both CT and 3D reconstructed images. Bowel obstruction due to an enlarged lesion was observed. The number of days since the date when peritoneal dissemination was first identified is annotated within the 3D images.

Nine months after the first surgery, the patient presented with abdominal pain, and two recurrent and enlarging lesions were identified in the abdominal cavity (**Fig. 3**). Despite repeated embolization of the intercostal, inferior phrenic, and inferior epigastric arteries, the lesion showed extraordinarily rapid growth. Due to marked abdominal distension and symptoms of bowel obstruction, surgical intervention was performed for diagnosis purposes and symptom relief. Intraoperatively, two soft peritoneal tumors were identified, one along the right paracolic gutter and the other in the pouch of Douglas. These lesions predominantly consisted of hematoma-like content, and macroscopic tumor resection with evacuation of the hematoma was performed as completely as feasible (**Fig. 2E**).

Histological examination revealed spindle cell components, leading to a diagnosis of an undifferentiated hepatic sarcoma (UHS) (**Fig. 2F**). Retrospective analysis confirmed the presence of similar cells within the granulation tissue under the capsule in the initial surgical specimen, establishing the diagnosis of peritoneal dissemination as a recurrence of the primary lesion (**Fig. 2D**). The immunohistochemical analysis revealed a high Ki-67 labeling index and p53 positivity, indicating high proliferative activity and potential genetic instability, and similar staining patterns were also observed in focal areas of the initial hepatic specimen. The lesion showed focal weak CD31 and smooth muscle actin (SMA) positivity, but was negative for CD34, Factor VIII, and erythroblast transformation-specific-related gene (ERG), making the diagnosis of angiosarcoma (AS) inconclusive, though Desmin positivity and weak SMA expression indicate partial myogenic differentiation. The negative staining of HepPar-1, Arginase-1, and Glypican-3 excludes the possibility of hepatocytic origin (**Fig. 2G** and **2H**). As the hepatic sarcoma was difficult to further classify, we requested a second opinion from a tertiary cancer center in Japan, and the diagnosis was consistent. Targeted sequencing revealed a DICER1 mutation in tumor cells, which has been reported to be associated with undifferentiated sarcoma (US).

## DISCUSSION

In this case, a hepatic lesion demonstrated imaging features resembling CEH, but subsequently exhibited an atypical progression, characterized by rupture and, after surgical resection, rapid interval growth of recurrence with intra-abdominal dissemination. Histopathological reexamination of the initial surgical specimen revealed focal spindle-shaped cells beneath the fibrous capsule, supporting the possibility that the lesion represented an underlying primary hepatic sarcoma that was underestimated at the initial evaluation.

CEH is a rare entity characterized by a long-standing hematoma encapsulated by fibrous tissue, which enlarges over time due to recurrent bleeding from capillaries within the chronically inflamed capsule.^1)^ According to a literature review^2–6)^ (1968 to March 2025), only 5 articles have reported hepatic CEH. All patients were Japanese, and hepatic CEH was rarely associated with previous trauma or bleeding predispositions. Rupture of hepatic CEH has not been reported to date, nor has PHS arising from hepatic CEH.

Five cases of AS^7)^ or a case of breast cancer^8)^ arising from CEH have highlighted the possibility that long-standing CEH may undergo neoplastic transformation. Chromosomal abnormalities involving chromosome 19—similar to those reported in both US and hepatic mesenchymal hamartoma^9)^—have also been described in CEH.^10)^ These overlapping genetic features may suggest a potential association between CEH and the subsequent development of US. Malignant cells were present within the fibrous capsule of CEH, though our attention tends to focus on the solid part of the lesion, as described in previous reports.^8)^

Reports of PHS in adults are even more limited. According to the WHO classification, sarcomas that show no specific line of differentiation despite immunohistochemical, morphological, and molecular analyses are categorized as USs.^11)^ Within this category, several morphologic patterns, including undifferentiated pleomorphic sarcoma (UPS), spindle cell, and round cell types, have been described.

In the present case, the tumor was highly undifferentiated and could not be assigned to a specific subtype based on immunohistochemical findings. Although the histologic features were compatible with UPS after excluding other specific sarcoma subtypes, classification as US is also appropriate. There is, however, no description of UHS or US of the liver in the WHO Digestive System Tumors classification.

Undifferentiated embryonal sarcoma (UES) is an important differential diagnosis among PHSs. It typically occurs in children and characteristically presents as a cystic-appearing hepatic mass on CT, with markedly high signal intensity on T2-weighted MRI reflecting abundant myxoid stroma. By contrast, the present case showed imaging features distinct from UES, including a thick fibrous capsule, rich arterial vascularity, and internal findings suggestive of mixed old and recent hemorrhage with hemosiderin deposition. Pathologically, UES is characterized by heterogeneous histology, often containing non-neoplastic bile ducts and hepatocytes, as well as PAS-positive giant tumor cells.^12)^ These histological features were not observed in the present case.

Another important differential diagnosis is hepatic angiosarcoma (AS), which is well known for its hemorrhagic tendency. Hemorrhage from peritoneal angiosarcomatosis has been reported as 27%,^13)^ and from the liver as 20%.^14)^ There are no specific tumor markers, and biopsy is generally not recommended given the risk of bleeding and dissemination. Typical imaging findings of AS are heterogeneous hyper vascular lesions enhanced progressively along early and delayed phase imaging,^15)^ which differed from the CEH-like encapsulated lesion observed in the present case. Complete surgical resection only provides the possibility of long-term survival, though hepatic sarcomas carry a high recurrence and poor prognosis across all histologic types. Interestingly, a few studies suggest that arterial embolization is of limited effectiveness for tumor enlargement or hemorrhage in hepatic sarcoma^16)^ and CEH.^4)^

The inconsistency and paucity of reports on UHS make it difficult to compare clinical courses and imaging features across published cases. It remains uncertain whether the PHS exhibited growth mimicking CEH, or whether a hepatic CEH underwent malignant transformation into sarcoma. Clinically, important lessons include the need to suspect malignant potential when rupture occurs, and the importance of carefully examining the subcapsular granulation tissue in lesions resembling CEH.

## CONCLUSIONS

This case highlights that primary hepatic lesions in an adult resembling CEH may represent or develop into US. Rupture and atypical progression should prompt suspicion for sarcoma and careful evaluation of subcapsular tissue.
